# The effect of education level on depressive symptoms in Chinese older adults–parallel mediating effects of economic security level and subjective memory ability

**DOI:** 10.1186/s12877-024-05233-5

**Published:** 2024-07-29

**Authors:** Ruonan Zhao, Jian Wang, Jiaxu Lou, Mei Liu, Jiahui Deng, Derong Huang, Huiling Fang

**Affiliations:** 1 Centre for Health Management and Policy Research, School of Public Health, Cheeloo College of Medicine, Shandong University, Jinan, 250012 China; 2 NHC KeyLab of Health Economics and Policy Research, Shandong University, Jinan, 250012 China; 3 Sun Yat-Sen Memorial Hospital, Sun Yat-Sen University, Guangzhou, Guangdong China

**Keywords:** Older people, Education level, Depressive symptoms, Economic security level, Subjective memory ability, Parallel mediation effect

## Abstract

**Background:**

Depression in older adults needs urgent attention. Increased education level may reduce depressive symptoms in older adults, and that economic security level and subjective memory ability may also have an impact on depressive symptoms in older adults, but the mechanisms between education level and depressive symptoms in older adults are unclear. This study endeavors to investigate the parallel mediating roles of economic security level and subjective memory ability between education level and depressive symptoms in older adults.

**Methods:**

A total of 4325 older adults people aged 60 years and above were selected from the China Family Panel Studies (CFPS) as the study population, and all data were analyzed using SPSS 25.0 software. Spearman correlation analysis was used to explore the correlation between the variables. Model 4 from the SPSS macro was used to assess the parallel mediating role of economic security level and subjective memory ability in the relationship between education level and depressive symptoms in older adults.

**Results:**

Education level, economic security level, and subjective memory ability were significantly associated with depressive symptoms in older adults (*p* < 0.01). Educational level was a negative predictor of depressive symptoms (β=-0.134, *P* < 0.001). Education level was a positive predictor of economic security level (β = 0.467, *P* < 0.001) and subjective memory ability (β = 0.224, *P* < 0.001). Education level, economic security level, and subjective memory ability were significant negative predictors of depressive symptoms (β= -0.039, *P* < 0.05; β= -0.122, *P* < 0.001; β= -0.169, *P* < 0.001). Education level influenced depressive symptoms through parallel mediating effects of economic security level and subjective memory ability, with mediating effects accounting for 42.70% and 28.30% of the total effect, respectively.

**Conclusions:**

Education level not only directly influences depressive symptoms in older adults, but also indirectly through the economic security level and subjective memory ability. Educational level can reduce depressive symptoms in older adults by increasing their economic security level and enhancing their subjective memory ability. The findings of this study emphasize the importance of improving the educational level of the population as it affects people’s mental health in old age.

## Introduction

Depression is a prevalent mental disorder among older adults. The global prevalence of depression in older adults is 28.4% [1], and the global prevalence of major depression reaches 13.3% [2]. The prevalence of depression among the older adults in China is high, reaching 25.55%, and the prevalence of depression tends to continue to increase over time [3]. The World Health Organization (WHO) ranked major depression as the third leading cause of the global burden of disease in 2008, and the disease is expected to rank first by 2030 [4]. Depression can be distressing for older adults, cause the breakdown of their families, and may lead to the worsening of existing illnesses and physical disability [5, 6]. Depression among the older adults should receive more attention in order to better achieve active aging and improve the physical health, mental health, and quality of life of the older adults.

Education level influences depressive symptoms in older adults, those with less education are at a higher risk of developing depressive symptoms [7–9]. Education represents people’s ability to access and use health information, and the level of education may have a greater impact on health than income or occupational status [10–12]. These results can be explained by the life course theory and the cumulative advantage/disadvantage theory. The life course theory, developed by Elder [13], emphasizes that the different life stages of a person are interconnected and that early life circumstances and experiences have long-term effects on the person [14, 15]. Cumulative advantage/disadvantage theory, on the other hand, suggests that early risk factors accumulate over the course of a person’s life and show their greatest impact later in life, meaning that early advantages or disadvantages are magnified over the life course [16, 17]. This shows that, a higher level of education is a lasting resource that produces advantages that accumulate over the course of life, increase happiness and joy in later life [18], and have a protective effect against depression throughout life [19]. Therefore, the effect of education level on depressive symptoms in older adults cannot be ignored.

Economic security is an important element of social security, retirement benefits, pension insurance and financial support from children are the main components of economic security for the older adults. Education level may affect the economic security level of the older adults. One study found that educated older adults being more likely to receive an occupational pension and receiving higher levels of benefits [20]. This may be due to the higher average income and social benefits of the educated population, and therefore their higher pension levels [21, 22]. Meanwhile, higher levels of pension can alleviate depression [23, 24], probably because pension receipt increases older adults’ confidence in the future [25, 26]. The marginal effect of pension receipt on enhancing mental health is stronger for older adults with poorer mental health [27], and pensions are most effective in alleviating depressive symptoms in older adults with low levels of education [28]. However, some scholars believe that a higher level of economic security may increase people’s depressive symptoms, which may be related to the specific countries and regions, cultural environment, sample population and other factors [29, 30].

Subjective memory is used to represent how individuals interpret, feel, or think about their memories, that is, the individual’s perception of memory performance [31]. The level of education may affect the subjective memory ability of the older adults. Education has a significant protective effect on memory capacity, with older adults with higher levels of education experiencing slower rates of memory decline [32]. Individuals with lower levels of education may be more likely to have memory deficits, and higher levels of education will reduce memory deficits associated with depressive symptoms [33]. Self-reported memory is important because it reflects the severity of depressive symptoms in older adults [34]. Lower memory ability at baseline survey was associated with worse levels of depressive symptoms at follow-up [35], suggesting that lower levels of memory ability may deepen depressive symptoms in older adults. In the older population, decreased subjective memory ability was associated with increased depression severity [36], educational interventions can improve memory loss in older adults and can alleviate their future depressive symptoms [37].

**Fig. 1:**
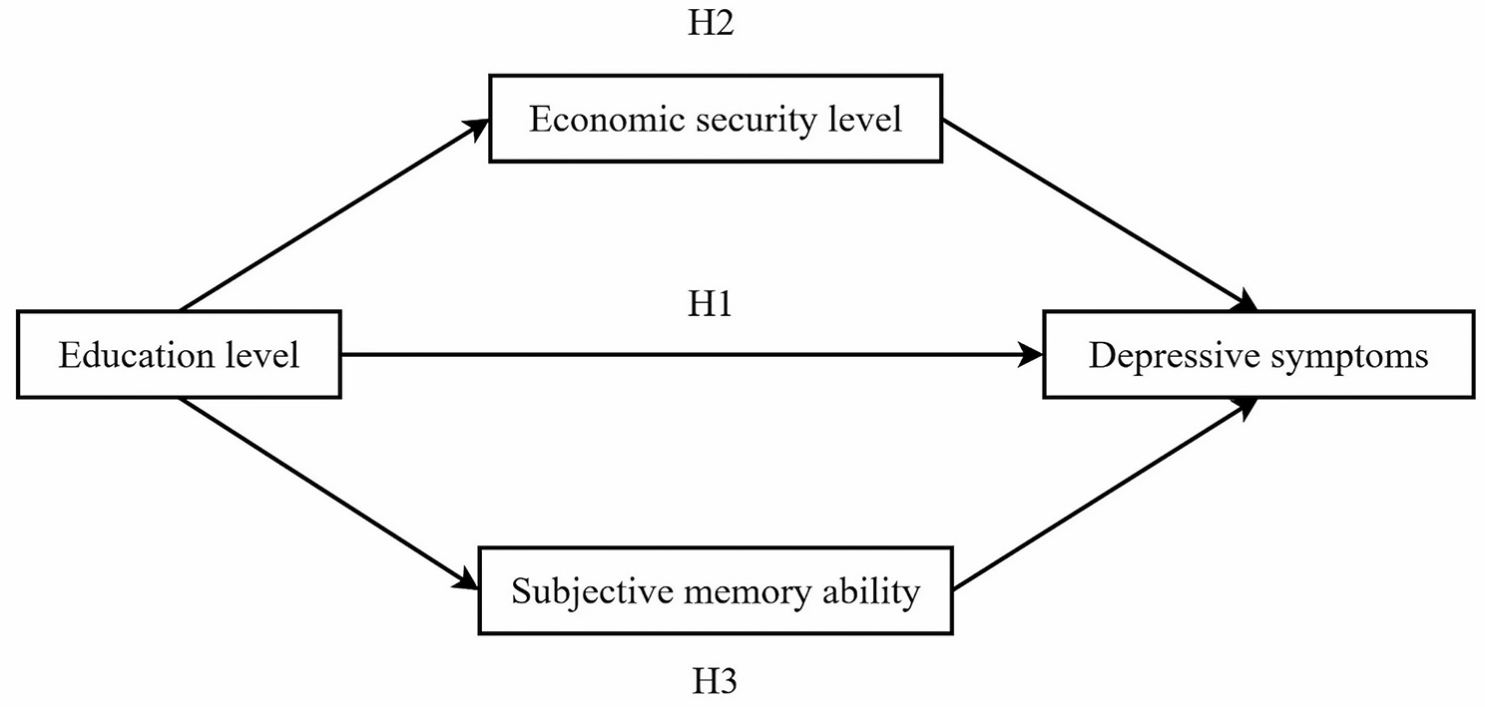
Parallel mediation model

Currently, although the relationship between education level and depressive symptoms in older adults has been investigated, the mechanisms between these two variables are unclear. No scholars have studied the relationship between education level, economic security level, subjective memory ability, and depressive symptoms in older adults. Therefore, the main purpose of this study was to explore the relationship between education level and depressive symptoms in older adults, to examine the parallel mediating role of economic security level and subjective memory ability in this relationship. The theoretical framework of this study is shown in Fig. 1, and we examined the following three hypotheses:

### Hypothesis 1

Educational level has a negative predictive effect on depressive symptoms in older adults.

### Hypothesis 2

Economic security level mediates the relationship between education level and depressive symptoms in older adults.

### Hypothesis 3

Subjective memory ability mediates the relationship between education level and depressive symptoms in older adults.

## Materials and methods

### Data source and sample selection

**Fig. 2:**
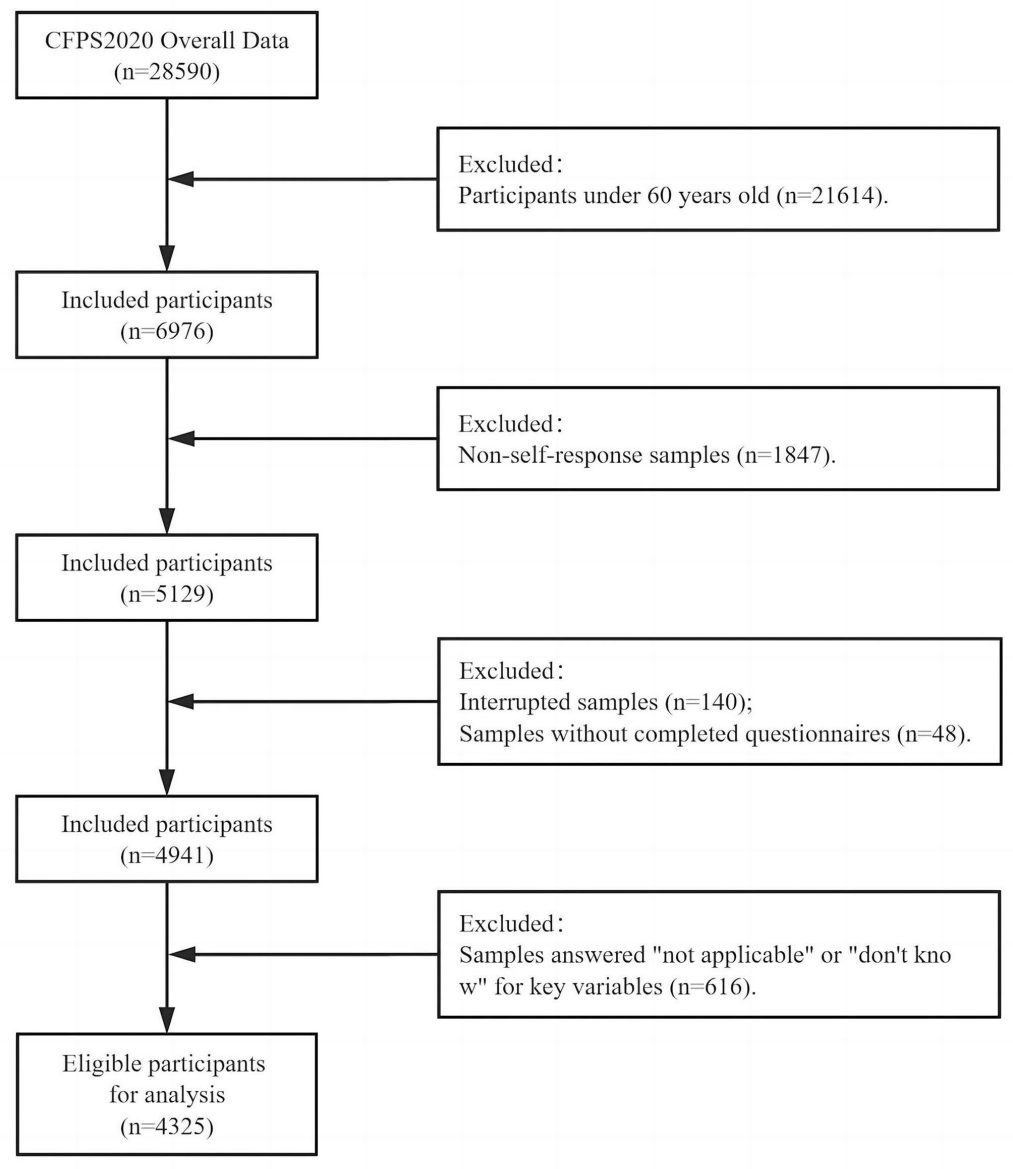
Selection of study subjects

The data for this study come from the China Family Panel Studies (CFPS), a biennial tracking survey conducted by the China Social Science Research Center at Peking University. The CFPS database collects data at the individual, household, and community levels, and investigates various aspects of Chinese residents’ economic activities, family relationships, and health status. CFPS officially launched the survey in 2010, with a sample covering 25 provinces/municipalities/autonomous regions in China. In this study, the contents of the individual-level questionnaire from the CFPS Round 5 survey in 2020 were selected for analysis, and older adults aged 60 years and above were chosen as the study population. The total number of samples in the 2020 CFPS database was 28,590, with 4,325 samples included after screening and the specific sample selection process is shown in Fig. 2.

### Measures

#### Education level

The independent variable studied in this paper is the educational level of the older adults, which is measured by the question in the questionnaire “What is the highest level of education you have completed (graduated)?” [22, 38]. According to the questionnaire responses, the answers were divided into five levels: “illiterate/semi-literate”, “elementary school”, “junior high school”, “high school”, and “college and above”, which were assigned a score of 1, 2, 3, 4, and 5, respectively, with higher scores indicating higher education levels [39, 40].

#### Depressive symptoms

The dependent variable studied in this paper was depressive symptoms in older adults. The 2020 CFPS questionnaire uses the CES-D8 to measure depressive symptoms in older adults [41]. The effectiveness of CES-D8 has been confirmed in previous studies [42]. SPSS analysis shows that Cronbach alpha of CES-D8 scale is 0.790, which indicates that the scale has good internal consistency reliability [43]. KMO value is 0.802, and *P* value in Bartlett’s Test of Sphericity is less than 0.001, which indicates that the scale has good validity [44]. The CES-D8 contains 8 questions related to depression, in which the respondents indicated the frequency of various feelings or behaviors in the past week according to their actual situation, and the answers were “hardly ever”, “some of the time”, “often”, and “most of the time”. Among these 8 questions, 2 positively stated questions scored 3 (hardly ever) to 0 (most of the time) and 6 negatively stated questions scored 0 (hardly ever) to 3 (most of the time) [8, 45]. The total score of the CES-D8 is 24, with higher scores indicating higher levels of depression; scale scores greater than or equal to 10 indicate a higher frequency of depressive symptoms in older adults [8, 46].

#### Economic security level

The level of economic security studied in this paper includes retirement benefits, pension insurance and financial support from children. The level of economic security is measured by the questionnaire questions “How much do you currently receive per month after tax, including your pension, various types of pension insurance and various allowances?” and “Please convert the gift into cash, how much did your children give you on average in cash in the past 6 months?” [47, 48]. The sum of the money answered in these two questions reflects the economic security level of the older adults.

#### Subjective memory ability

The subjective memory ability of older adults was measured by the questionnaire “How many major events that happened to you in the last week can you remember?” [49, 50]. The answers to this question included “can barely remember”, “can only remember a few”, “can remember half”, “can remember most”, and “can remember completely”, and were assigned a score of 1, 2, 3, 4, and 5, with higher scores indicating better subjective memory ability [51, 52].

#### Covariates

In this study, a number of confounding factors associated with depressive symptoms in older adults were selected as covariates [38, 39, 46, 53], including: age, gender, marital status, smoking status, alcohol consumption, neighborhood trust, and kinship. These variables were all measured by the 2020 CFPS individual-level questionnaire.

### Statistical analyses

All data were analyzed by SPSS 25.0 software. Firstly, we launched a descriptive statistical analysis of the main study variables. Secondly, there was non-normally distributed data in the study variables, so we used spearman correlation analysis to explore the correlations between education level, economic security level, subjective memory ability, and depressive symptoms in older adults. Finally, we used Model 4 from the SPSS macro developed by Hayes [54–56] to assess the parallel mediating role of economic security level and subjective memory ability in the relationship between education level and depressive symptoms in older adults. Based on a random sample of 5000, a bootstrapping method was used to estimate 95% confidence intervals to test the significance of the mediating effect. The results were considered statistically significant when the 95% confidence interval did not contain 0 [57].

## Results

### Primary analyses

Table 1 is the descriptive statistical analysis results of the research population. Among the 4325 respondents included in this study, 2224 (51.4%) were men and 2101 (48.6%) were women. Respondents’ ages ranged from 60 to 95 years, with a mean age of 68.3 years (SD = 5.8). In terms of education level, 1,718 (39.7%) were illiterate or semi-literate, 970 (22.4%) had elementary school education, 942 (21.8%) had junior high school education, 563 (13.0%) had high school education, and 132 (3.1%) had college education and above. In terms of depressive symptoms, there were 792 (18.3%) older adults with a higher frequency of depressive symptoms. The median economic security level for the older adults is $125.8/month and the interquartile range is $329.6/month. Regarding the subjective memory ability of the older adults, 1268 (29.3%) could barely remember the main events that happened within a week, 761 (17.6%) could only remember a few, 1175 (27.2%) could remember half, 647 (15.0%) could remember most, and 474 (11.0%) could remember completely.

**Table 1:** Descriptive statistics (sample *n* = 4325)

Variables	Participants assessed
Gender, n (%)	
Female	2101 (48.6)
Male	2224 (51.4)
Age, mean (SD)	68.3 (5.8)
Marital status, n (%)	
Unmarried	25 (0.6)
Married	3591 (83.0)
Cohabit	19 (0.4)
Divorced	54 (1.2)
Widowed	636 (14.7)
Smoking status, n (%)	
No	3139 (72.6)
Yes	1186 (27.4)
Alcohol consumption, n (%)	
No	3632 (84.0)
Yes	693 (16.0)
Neighborhood trust, n (%)	
Low	293 (6.8)
Medium	1845 (42.7)
High	2187 (50.6)
Kinship, n (%)	
Poor	253 (5.8)
Moderate	1545 (35.7)
Good	2527 (58.4)
Education level, n (%)	
Illiterate or semi-literate	1718 (39.7)
Elementary school education	970 (22.4)
Junior high school education	942 (21.8)
High school education	563 (13.0)
College education and above	132 (3.1)
Depressive symptoms, n (%)	
High frequency	792 (18.3)
Low frequency	3533 (81.7)
Economic security level, Me(IQR)	125.8 (329.6)
Subjective memory ability, n (%)	
Barely remember the main events	1268 (29.3)
Bnly remember a few	761 (17.6)
Remember half	1175 (27.2)
Remember most	647 (15.0)
Remember completely	474 (11.0)

The results of the correlation analysis are shown in Table 2. Educational level was significantly positively correlated with economic security level (ρ=-0.391, *P* < 0.01) and subjective memory ability (ρ = 0.241, *P* < 0.01), and significantly negatively correlated with depressive symptoms (ρ=-0.175, *P* < 0.01); depressive symptoms were significantly negatively correlated with economic security level (ρ=-0.216, *P* < 0.01) and subjective memory ability (ρ=-0.239, *P* < 0.01); economic security level was significantly positively correlated with subjective memory ability (ρ = 0.210, *P* < 0.01).

**Table 2:** Correlation analysis results

Variable	Education Level	Depressive symptoms	Economic security level	Subjective memory ability
Education Level	1	-0.175^**^	0.391^**^	0.241^**^
Depressive symptoms		1	-0.216^**^	-0.239^**^
Economic security level			1	0.210^**^
Subjective memory ability				1

**P < 0.01.

### Parallel mediation analysis results

**Fig. 3:**
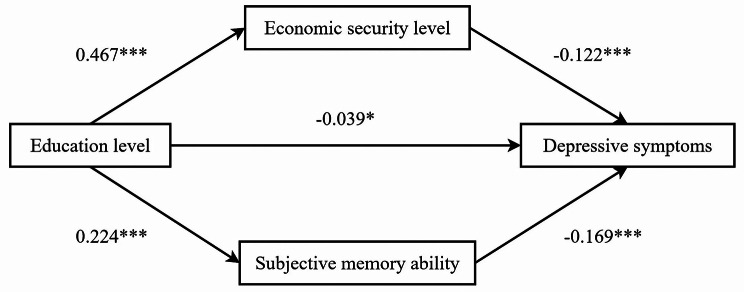
Parallel mediating roles of economic security level and subjective memory ability between education level and depressive symptoms in older adults. ***P < 0.001, *P < 0.05

Table 3; Fig. 3 show the results of regression analysis in the mediating effect model. Under the control of gender, age, marital status, smoking status, alcohol consumption, neighborhood trust and kinship, the parallel mediating effect of economic security level and subjective memory ability between education level and depression symptoms of the older adults was tested. The results of model 1 show that education level has a significant negative predictive effect on depressive symptoms (β=-0.134, *P* < 0.001). The results of model 2 and model 3 show that, education level was a positive predictor of economic security level (β = 0.467, *P* < 0.001) and subjective memory ability (β = 0.224, *P* < 0.001). Model 4 adds education level, economic security level, subjective memory ability and depressive symptoms to the regression model. it was found that education level, economic security level, and subjective memory ability were significant negative predictors of depressive symptoms (β= -0.039, *P* < 0.05; β= -0.122, *P* < 0.001; β= -0.169, *P* < 0.001).

**Table 3:** Results of mediating-effect model

	Model 1: Depressive symptoms		Model 2: Economic security level		Model 3: Subjective memory ability		Model 4: Depressive symptoms	
	β	t	β	t	β	t	β	t
Education Level	-0.134	-8.703***	0.467	32.978***	0.224	14.511***	-0.039	-2.288*
Economic security level							-0.122	-7.508***
Subjective memory ability							-0.169	-11.314***
Gender	-0.114	-6.173***	-0.023	-1.359	0.043	2.352*	-0.109	-6.064***
Age	-0.022	-1.452	0.159	11.233***	0.014	0.879	-0.001	-0.037
Marital status	0.093	6.010***	-0.044	-3.122**	0.007	0.461	0.088	5.867***
Smoking status	0.050	2.897**	-0.031	-1.925	0.009	0.525	0.048	2.834**
Alcohol consumption	-0.046	-2.922**	0.028	1.953	0.049	3.105**	-0.034	-2.223*
Neighborhood trust	-0.085	-5.398***	-0.028	-1.913	-0.001	-0.077	-0.089	-5.759***
Kinship	-0.105	-6.646***	0.026	1.794	0.087	5.462***	-0.087	-5.628***
Constant	10.281	12.065***	-3570.834	-10.152***	1.307	5.096***	10.043	11.870***
R^2^	0.079		0.219		0.070		0.122	
F	45.967***		151.371***		40.327***		59.733***	

***P < 0.001, **P < 0.01, *P < 0.05

Table 4 shows the results of the parallel mediated effects test with unstandardized effect values. The upper and lower limits of bootstrap 95% confidence intervals for the direct effect of education level on depressive symptoms and the mediating effect of economic security level and subjective memory ability did not include 0. This suggests that education level not only has a direct effect on depressive symptoms, but also has an effect through a parallel mediating effect of economic security level and subjective memory ability. The direct effect of education level on depression level was − 0.147, accounting for 28.99% of the total effect. The mediating effects of economic security level and subjective memory ability were − 0.217 and − 0.144, accounting for 42.70% and 28.30% of the total effect, respectively. The total mediating effect was − 0.361, accounting for 71.01% of the total effect.

**Table 4:** Total, direct and mediated effects

	Effect	Boot SE	Boot LL CI	Boot UL CI	Effect size
Total effect	-0.508	0.058	-0.622	-0.393	
Direct effect	-0.147	0.064	-0.273	-0.021	28.99%
Total mediating effect	-0.361	0.029	-0.418	-0.305	71.01%
Mediating effect of economic security level	-0.217	0.026	-0.269	-0.167	42.70%
Mediating effect of subjective memory ability	-0.144	0.016	-0.176	-0.113	28.30%

## Discussion

Based on the 2020 CFPS database, this study explored the parallel mediating role of economic security level and subjective memory ability between education level and depressive symptoms in older adults. The results of the correlation analysis showed that the education level was positively correlated with the economic security level and subjective memory ability, and negatively correlated with depressive symptoms. The economic security level and subjective memory ability were negatively correlated with depressive symptoms. The results of parallel mediation tests showed that the economic security level and subjective memory ability mediated the relationship between education level and depressive symptoms in older adults. The education level may reduce depressive symptoms in older adults by increasing their economic security level and enhancing their subjective memory ability.

At present, the overall education level of Chinese elder people is low, nearly 40% of them are illiterate or semi-literate, and there is a large gap in education level among them. From the perspective of life course theory and cumulative advantage/disadvantage theory, these gaps in educational attainment during student years may affect people’s future work and social interactions, which in turn may have an impact on depressive symptoms in old age [10, 58]. The results of this study show that the mental health of the older adults in China is generally good. There are 18.3% older adults with high incidence of depressive symptoms, and some older adults score close to 24 on CES-D8 scale, which shows that the depression of these people needs urgent attention. The 18.3% prevalence of depressive symptoms among older adults in China is at an intermediate level. The older adults in different countries have different levels of depression, which may be related to the national conditions and social environment. The prevalence of depressive symptoms in adults in the United States is 7.3% [59], and the proportion of older adults aged 60 and over in the United States who are diagnosed with depression is 8.18% [60]. The prevalence of depression increases with age, reaching 13.9% among people in their 60s and 70s and 18.4% among people in their 80s or above in Korea [61]. The proportion of older adults with clinically significant depressive symptoms in Biljand, Iran is 19.94% [62]. The prevalence of depressive symptoms among the older adults in Vietnam is 20.2% [63]. Therefore, the 18.3% depression rate of the older adults in China needs attention, and we should actively find measures to reduce the depression of the older adults.

The present study explored the relationship between education level and depressive symptoms in older adults, and the results showed a significant negative predictive effect of education level on depressive symptoms in older adults, which is consistent with previous studies [7, 9, 64], and hypothesis 1 was supported. Education is an important component of socioeconomic status [65], and the results of this study suggest that there may be potential clinical implications of changing educational patterns. Some scholars have considered the dual effect of education level and family background on depressive symptoms in older adults, and they found that individuals with low education from poor family backgrounds exhibited the highest levels of depressive symptoms [66], which reminds the government to pay special attention to the depressive status of older adults with low education levels. As indicated by the cumulative advantage/disadvantage theory, although high levels of education have a weaker protective effect on depressive symptoms in early adulthood, they have a stronger protective effect on depressive symptoms in old age, and the effect of education level on depression increases over time [19]. Higher levels of education can not only prevent major depressive disorder, but also change its presentation to a more anxious phenotype [40]. The educational level of older adults may also affect the educational level of their children, which in turn may affect their children’s intergenerational support and ultimately their own depressive symptoms [67].

There has been no study that combines retirement benefits, pension insurance and financial support from children to explore the mediating role of the economic security level in the relationship between education level and depressive symptoms in older adults. The mediating role of economic factors between education level and depressive symptoms in older adults has been explored: the study of Lingli Li suggested that family economic factors play a crucial role between these two factors [39], the study of Xiwu Xu showed a mediating role of economic level between education and depression [38], Sandro Sperandei suggested a mediating role of income level between these two factors [68], and Yaolin Pei demonstrated the mediating role of children’s financial support between education and depressive symptoms in older adults [67]. This study found that the economic security level plays a mediating role between education level and depressive symptoms in older adults, with a mediating effect of 42.70%, and hypothesis 2 was supported. Higher levels of education increase the level of financial security, which in turn reduces depressive symptoms in older adults. Life course theory and cumulative advantage/disadvantage theory suggest that the different stages of a person’s life are interconnected. The early education level may influence people’s employment and income levels [11], and even their perceptions of medical care and pension insurance choices [21], which in turn increases the gap in the economic security level in later life. In contrast, higher economic security levels can reduce depressive symptoms by alleviating financial stress and increasing confidence in the future among older adults [23, 25].

In addition to the economic security level, this study also explored the mediating role of subjective memory ability. Subjective memory ability mediated the relationship between education level and depressive symptoms in older adults, with a mediating effect of 28.30%, and hypothesis 3 was supported. Higher education level enhances subjective memory ability, which in turn reduces depressive symptoms in older adults, which is similar to Xiwu Xu’s findings on the mediating role of cognitive level in older adults [38]. A possible biological explanation is that higher levels of education may improve cognition in the older adults, which in turn inhibits the expression of inflammatory cytokines and ultimately reduces the incidence of depression [39]. Based on cumulative advantage theory, early school education has exercised people’s cognitive and memory abilities [69, 70], and these mindfulness exercises may influence people’s memory and cognitive abilities in their later life. In contrast, higher subjective memory ability can guarantee the living standards and well-being of older adults [34], which in turn reduces depressive symptoms in older adults. The results of the parallel mediation analysis showed that in terms of the effect of education level on depressive symptoms, the economic security level showed a greater mediation effect than subjective memory ability, and higher education level was more able to reduce depressive symptoms in older adults by increasing the their economic security level.

The main contribution of this study is to further clarify the mechanism between education level and depressive symptoms in older adults. It also reveals the critical role of the economic security level and subjective memory ability in this relationship, which enriches the research on the relationship between education level and depressive symptoms in older adults. The parallel mediation model of this study is based on life course theory, cumulative advantage/disadvantage theory and previous empirical research. The total mediation effect of economic security level and subjective memory ability is -0.361, and the effect ratio is 71.01%. From this, it can be seen that the level of economic security and subjective memory ability play a strong intermediary role between education level and depression symptoms of the older adults, so the results of parallel intermediary analysis are scientific and reasonable. In addition, the baseline sample of CFPS database used in this study covers 25 provinces/municipalities/autonomous regions in China, representing 95% of the population in China, and the sample is highly representative [71]. This indicates that the results of this study are basically in line with the actual situation of Chinese older adults, and the findings are of practical significance for reducing the level of depression among older adults, as well as for research on depression among older adults in other countries.

However, there are also some limitations of this study. Firstly, our study was cross-sectional in design, which limits our interpretation of the causal relationship between education level and depressive symptoms in older adults. Future scholars can verify the mediating role of the economic security level and subjective memory ability through cohort studies, and can further consider the heterogeneity in terms of urban and rural areas, males and females. Secondly, due to data limitations, we chose subjective memory ability rather than objective cognitive test results as a mediating variable, which may have led to bias in the study results. Future scholars can use the scale test results related to older adults’ cognitive abilities to validate the findings of this study. Finally, we considered only two mediating variables. Future studies can continue to include more specific mediating variables to explore the relationship between education level and depressive symptoms in older adults, such as lifestyle and physical health. Scholars can also explore the intermediary role between education level and depression symptoms of the older adults based on the national conditions and population structure of different countries.

## Conclusion

The results of this study suggest that education level not only directly influences depressive symptoms in older adults, but also indirectly through the economic security level and subjective memory ability. Education level may reduce depression in older adults by increasing their economic security level and enhancing their subjective memory ability. In this era of general improvement in people’s education level, it reminds policy makers to pay attention not only to people’s overall education level, but also to education equity. Making a balanced distribution of educational resources among regions can, to a certain extent, promote mental health equity in people’s old age.

## Data Availability

The raw data is publicly available at https://www.isss.pku.edu.cn/cfps/index.htm.
